# Dizeez: An Online Game for Human Gene-Disease Annotation

**DOI:** 10.1371/journal.pone.0071171

**Published:** 2013-08-07

**Authors:** Salvatore Loguercio, Benjamin M. Good, Andrew I. Su

**Affiliations:** Department of Molecular and Experimental Medicine, The Scripps Research Institute, La Jolla, California, United States of America; King Abdullah University of Science and Technology, Saudi Arabia

## Abstract

Structured gene annotations are a foundation upon which many bioinformatics and statistical analyses are built. However the structured annotations available in public databases are a sparse representation of biological knowledge as a whole. The rate of biomedical data generation is such that centralized biocuration efforts struggle to keep up. New models for gene annotation need to be explored that expand the pace at which we are able to structure biomedical knowledge. Recently, online games have emerged as an effective way to recruit, engage and organize large numbers of volunteers to help address difficult biological challenges. For example, games have been successfully developed for protein folding (Foldit), multiple sequence alignment (Phylo) and RNA structure design (EteRNA). Here we present Dizeez, a simple online game built with the purpose of structuring knowledge of gene-disease associations. Preliminary results from game play online and at scientific conferences suggest that Dizeez is producing valid gene-disease annotations not yet present in any public database. These early results provide a basic proof of principle that online games can be successfully applied to the challenge of gene annotation. Dizeez is available at http://genegames.org.

## Introduction

Using the tools of high-throughput biology, scientists can quickly identify long lists of candidate genes that differ between two experimental conditions. Structured gene annotations are essential to interpret these gene lists and to discover fundamental properties like gene function and disease relevance. Gene set enrichment, pathway modeling, and cross-genome comparisons are just a few of the analyses that depend on structured gene annotations [1], [2]. The importance of methods like these will only grow as the rate of genomic data generation increases.

However, the representation of gene annotations is quite sparse. For example, at the time of writing only 57% of human protein-coding genes have two or more human-curated GO annotations. Structured data for diseases are even less complete. These gaps are, at least in part, due to inefficiencies in the translation of scientific knowledge into structured annotations. Currently, we rely on a few large biocuration groups to translate all of the peer-reviewed literature into structured annotations. However, these centralized efforts simply cannot keep up with the rate of biomedical data generation. It has been estimated that the current manual curation processes will take far too long to complete the annotations of even just the most important model organisms [3]. The biocuration community itself has noted that “the exponential growth in the amount of biological data means that revolutionary measures are needed for data management, analysis and accessibility” [4].

Recently, “crowdsourcing” has emerged as a complementary approach that directly harnesses the collaborative efforts of large communities of people. This principle, which has been the foundation of many successful web-based applications, has also been applied to scientific challenges of massive scale. For example, the Galaxy Zoo initiative enables citizen astronomers to classify galaxies in large sets of celestial images [5], and the Gene Wiki project engages the research community to create a gene-specific review article for every human gene [6]. Similar initiatives have emerged for RNA families [7] and biological pathways [8].

One emerging trend among crowdsourcing initiatives is the use of games as a mechanism to attract contributors - in particular, ‘games with a purpose’ (GWAPs) that collaboratively harness gamer's time and energy for productive ends. One of the first GWAPs, called the “ESP Game”, had the ambitious goal of tagging all online images with informative keywords. It resulted in 50 million labels produced by more than 200,000 players [9]. Similarly successful games were later developed to annotate music, text, and videos [10], [11]. Online games have also been shown to be an effective collaborative platform to address challenging biological problems. For example, the Foldit game (http://fold.it) [12] addresses a fundamental biomedical challenge: computational protein folding. It has harnessed the efforts of over 300,000 gamers to predict protein structure from primary sequence, to provide accurate structural models that led to the crystal structure of a previously intractable retroviral protease, and to design new protein folding strategies and algorithms [13]. Other examples include Phylo for improving multiple sequence alignments [14] and EteRNA for designing RNA structures (http://eterna.cmu.edu).

Here we introduce Dizeez, an online game aimed at cataloging gene-disease associations that are well-established in the literature but not yet reflected in structured annotation databases. We provide preliminary results from game play online and at scientific conferences. These data suggest that even after limited game play, novel gene-disease annotations can be mined from game playing logs.

## Methods

**Figure 1:**
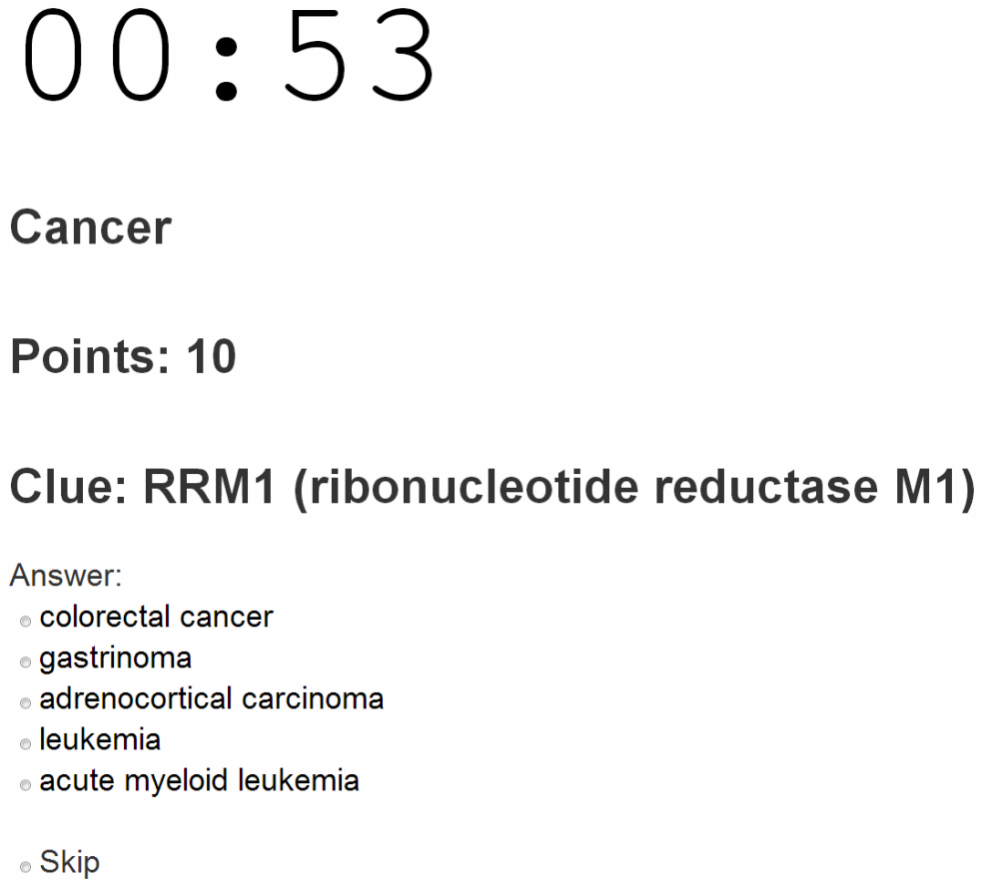
Dizeez - main game interface.

Dizeez is a multiple choice quiz where the player is presented with a disease drawn from the Human Disease Ontology [15] (the “Clue”) and a multiple-choice selector with five genes, only one of which has prior evidence linking it to the Clue disease (**Figure 1**). We used a set of 3,439 candidate gene-disease links mined from the Gene Wiki [16] as the input data set for the Dizeez game. The game randomly selects one of these links, and hides the disease among four randomly chosen diseases (the four random diseases were sampled at the frequency with which they appeared in the input Gene Wiki dataset). If the player correctly guesses the known disease from the list of five possible diseases, they receive points. Regardless, all player answers are logged by the system as gene-disease “assertions”. Players are challenged to accumulate as many points as possible in a one-minute round.

**Figure 2:**
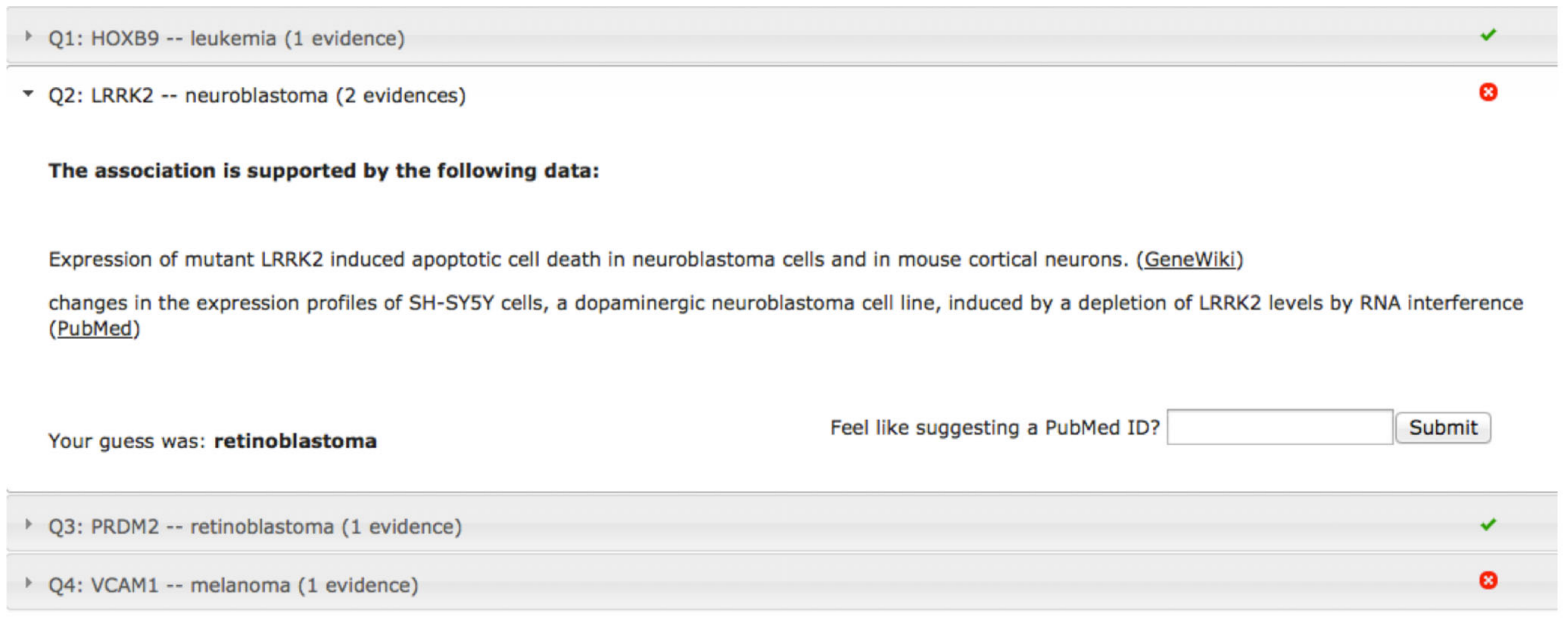
Dizeez - game review interface.

To match game players to genes about which they are likely to have first-hand knowledge, Dizeez allows players to select a specific disease area (e.g., cancer, metabolism, immunology) or a specific protein family (kinases, proteases, GPCRs). At the end of each round, players can review a recap of all questions that shows supporting evidence (based on text extracted from the Gene Wiki and GeneRIFs) for each gene-disease association recorded in a game. Users can review the game log and even suggest new evidence for gene-disease associations (**Figure 2**).

As mentioned above, every player Guess in the game can be interpreted as an assertion of a putative gene association between the Clue (gene) and the Guesses (diseases). Candidate annotations that are independently reported across multiple players will obtain the highest confidence scores, according to the value of independent replication. This concept of replication or ‘voting’ is used extensively to improve results in related crowdsourcing initiatives [5], [9]. In other contexts this confidence is referred to as ‘inter-annotator agreement’ and is used to assess the quality of professional annotations [17].

## Results

We released Dizeez to the community in December 2011; publicizing its existence through our lab blog, twitter account and game play at a scientific conference. Within nine months, 1,045 games had been played to completion by over 230 unique individuals (as estimated from the number of unique IP addresses recorded during gameplay). Overall, players provided 8,525 guesses resulting in 6,941 unique gene-disease assertions. A total of 2,188 out of these 6,941 assertions were previously annotated in OMIM (via Human Disease Ontology cross references to OMIM), 3,384 were previously annotated in PharmGKB and 1,448 were previously annotated in the Gene Wiki [16]. A total of 2,137 gene-disease assertions were not found in any of these gene-disease databases.

**Figure 3:**
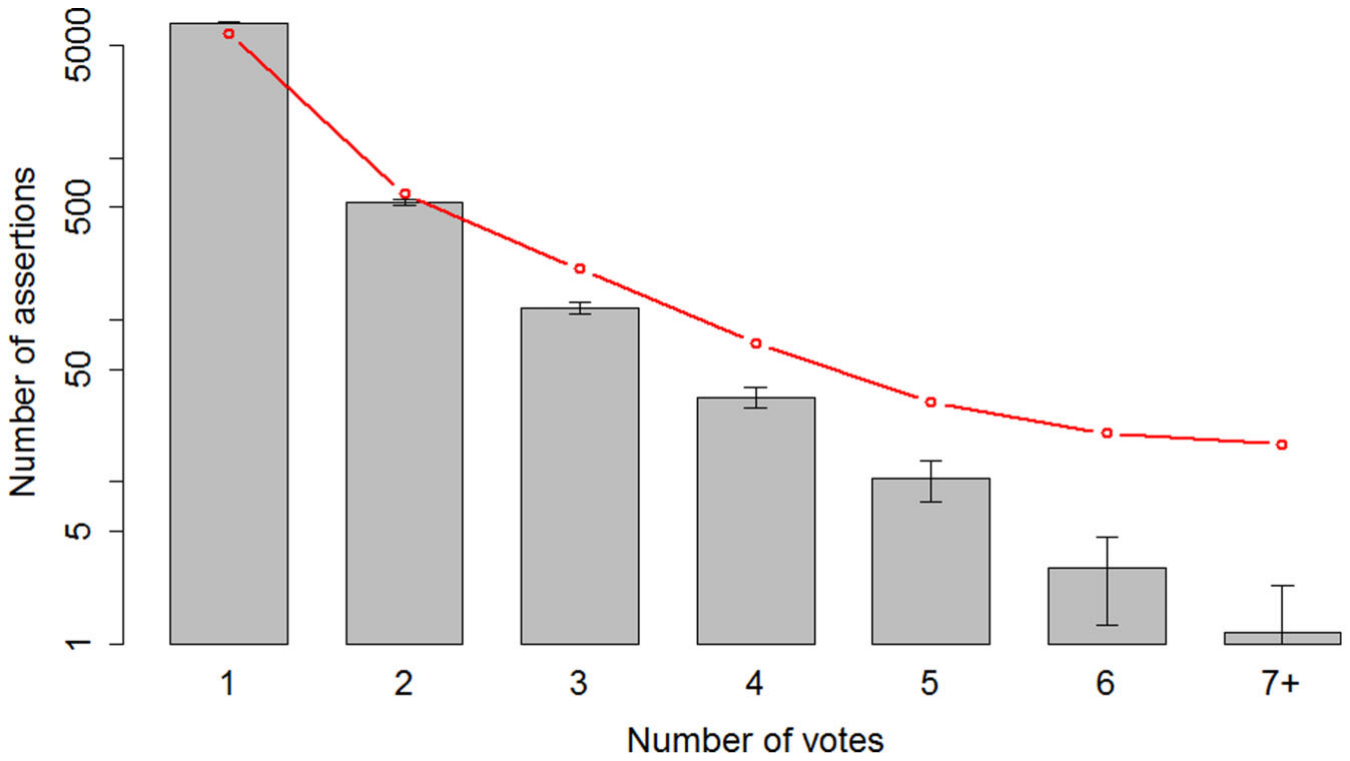
Number of Gene-Disease assertions vs. number of votes, for real- and random gameplay. The vertical axis represents the number of associations collected during game play (log scale). Red line: real gameplay. Grey bars: mean number of associations after 100 randomizations, with associated standard deviation. ‘7+’ indicates the sum of associations collected with a number of votes equal or greater than 7.

Clearly, each individual gene-disease assertion is not equally valuable. We hypothesized that assertions that were replicated across many games and game players were more likely to be valid. For example,among the gene-disease assertions provided most often by game players, we found 17 associations occurring 7 or more times (**Table 1**). We compared these data to a simulation in which game players randomly selected a disease from the five presented options (**Figure 3**). The simulation showed that the number of votes per assertion was significantly higher than random at replication values greater than one. This demonstrates that the observed replication is unlikely to be the mere result of chance association.

**Table 1:** Gene-disease associations provided seven or more times in Dizeez.

# Votes	Gene Symbol	Gene Name	Disease	OMIM	PharmGKB	DGA	PubMed (PMID)
11	NBPF3	neuroblastoma breakpoint family, 3	neuroblastoma	No	No	No	19536264, 18493581
11	SOX8	SRY (sex determining region Y)-box 8	mental retardation	No	No	No	18076105, 10684944
9	ABL1	c-abl oncogene 1, non-receptor tyrosine kinase	leukemia	No	Yes	Yes	3313010, 6308652
9	SSX1	Synovial sarcoma, X breakpoint 1	synovial sarcoma	No	No	Yes	12037676, 12696068
8	APC	Adenomatous polyposis coli	colorectal cancer	Yes	Yes	Yes	10737795, 2188735
8	FES	Feline sarcoma oncogene	sarcoma	No	No	Yes	—
8	RBP3	Retinol binding protein 3, interstitial	retinoblastoma	No	No	No	—
8	GAST	Gastrin	gastrinoma	No	No	No	7439637, 5648596
8	DCC	Deleted in colorectal carcinoma	colorectal cancer	No	No	Yes	22876889, 22920895
8	MAP3K5	mitogen-activated protein kinase kinase kinase 5	Cancer	No	No	Yes	22197930, 22723553
7	RB1	retinoblastoma 1	retinoblastoma	Yes	No	Yes	2877398, 3823889
7	RET	ret proto-oncogene	Cancer	No	Yes	Yes	23170308, 23150706
7	MLL3	myeloid/lymphoid or mixed-lineage leukemia 3	leukemia	No	No	No	—
7	BACE2	beta-site APP-cleaving enzyme 2	Alzheimer's disease	No	No	Yes	22074738, 22044119
7	GTF2I	general transcription factor IIi	developmental disorder	No	No	No	19897463, 20956978
7	MFI2	antigen p97 (melanoma associated)	melanoma	No	No	Yes	20935638
7	KRAS	v-Ki-ras2 Kirsten rat sarcoma viral oncogene homolog	colorectal cancer	No	Yes	Yes	23188063, 23182985

All of the associations reported in Table 1 were previously known in our Gene Wiki-derived data set of annotations. Given that this data set was the source of the “right answers”, finding these annotations reproduced by game play data served as a positive control. Among this set of candidate annotations, the gene WRN (“Werner syndrome, RecQ helicase-like”) was linked to the disease Werner syndrome and the gene CRYGC (“crystallin, gamma C”) was linked to cataracts. We evaluated these 17 candidate annotations through manual literature search in PubMed, and successfully found evidence for all but three of them with an overall specificity of 82%.

Interestingly, the three false positives were all cases where the gene name seemed to suggest a disease association that was not substantiated in the literature. For example, the *FES* gene (“Feline sarcoma oncogene”) in humans has no known role in sarcoma. Similarly, there is no evidence that *RBP3* (“Retinol binding protein 3, interstitial”) is involved in retinoblastoma, nor that *MLL3* (“myeloid/lymphoid or mixed-lineage leukemia 3”) is involved in leukemia. This observation suggests that human game players are susceptible to some of the same kinds of errors that text-mining systems suffer from. In future iterations of the game, Dizeez will focus on introducing reward mechanisms for players who use more context than simply a gene's name when making gene-disease assertions. Next, we mined Dizeez game logs for novel gene-disease links that were well established in the literature and also did not appear in our Gene Wiki-derived input data set. In short, these assertions corresponded to “wrong” answers that were repeated multiple times by multiple game players. There were 6 such assertions that were provided by players four or more times (**Table 2**). Through manual validation in the primary literature, we could find evidence for those assertions in 5 of those 6 cases. In the lone false positive, game players suggested that the *HTT* gene (“huntingtin”), which is known to be implicated in Huntington's disease, was involved in Alzheimer's disease. Since both diseases involve aberrant protein aggregation resulting in progressive cognitive dysfunction, this assertion by game players was plausible, though ultimately not supported by conclusive research.

**Table 2:** Gene-disease associations provided four or more times in Dizeez and not found in Gene Wiki.

# Votes	Gene Symbol	Gene Name	Disease	OMIM	PharmGKB	DGA	PubMed (PMID)
6	HTT	huntingtin	Alzheimer's disease	No	No	No	–
5	BCL2	B-cell CLL/lymphoma 2	leukemia	No	No	Yes	23118966, 23114648
5	MECOM	MDS1 and EVI1 complex locus	sarcoma	No	No	No	18206536
5	PRDM2	PR domain containing 2	neuroblastoma	No	No	No	20878080, 18819740
4	AVPR1A	arginine vasopressin receptor 1A	Alzheimer's disease	No	No	No	21115064
4	ATF7	activating transcription factor 7	Cancer	No	No	No	22260696, 17309674

**Figure 4:**
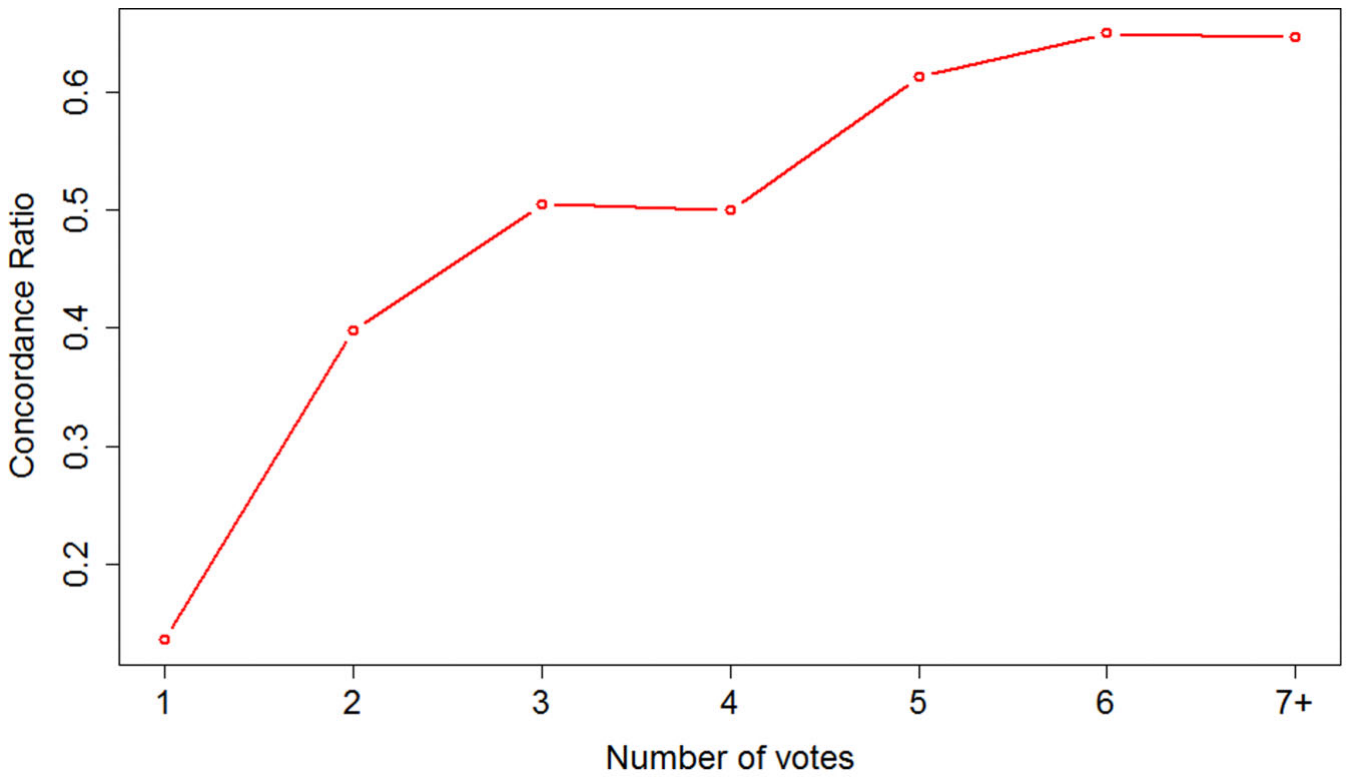
Concordance between Dizeez-mined associations and Disease and Gene Annotations database. The ‘concordance ratio’ on the vertical axis is the ratio between the associations supported by DGA and the total number of associations for a given number of votes. ‘7+’ indicates the sum of associations collected with a number of votes between 7 and 11.

Finally, we examined the assumption that the number of independent assertions (“votes”) for each candidate gene-disease association correlates with the likelihood that the association is supported by the literature (**Figure 4**). To do this for all of the collected assertions, we compared them to the recently published Disease and Gene Annotations database (DGA) [18].As the number of votes increases from 1 to 7 and above, the concordance between the Dizeez-mined associations and the DGA associations steadily increases from 0.14 (812/5985) to 0.65 (11/17). The number of assertions to pass each vote threshold steadily decreases from 5,985 with only one vote down to just 13 assertions at the 6-vote level (Figure 3). As such, the number of votes per assertion provides a mechanism for tuning the system towards either higher recall (low vote requirement) or high precision (high vote requirement).

While we suggest that DGA provides reasonably good coverage of known gene-disease associations and is thus a useful point of reference for comparison, it is by no means exhaustive and thus we would not expect to reach 100% concordance. Many valid associations captured by Dizeez may not be present in DGA. For example, the Dizeez-generated and manually-validated associations between SOX8 and mental retardation and between NBPF3 and Neuroblastoma (Table 1) are not present in the DGA.

## Discussion

A common concern raised against any form of crowdsourcing in a scientific context is that the ‘crowd’ will not produce high quality data. While it may be true that the average participant in these systems – whether as a player of Dizeez or an editor of the Gene Wiki – may not contribute data of equal quality to that produced by a trained professional, the *aggregated* labor of many participants can produce useful, high quality resources. This step of aggregation – of filtering and combining contributions from multiple diverse sources – distinguishes crowdsourcing efforts from traditional, professional systems that assume each individual contribution is correct from the outset.

The early results from Dizeez show two key things: 1) a very simple online game can produce a large number of gene-disease associations in a relatively short amount of time and 2) a simple voting system can easily and reliably identify the high quality gene-disease associations within the set contributed by the game players.

In future work, we intend to refine the aggregation system by weighting the votes from different players based on their ability to reproduce known gene-disease associations during game play. In addition, we could build upon the current ‘game-review’ functionality with the purpose of educating players about published information about genes and diseases.

One fundamental weakness of Dizeez is that players are “punished” when they add potentially novel associations. That is, there is no game reward when they add a novel, true annotation. To better the game mechanic to encourage contributions of novel annotations, we are also exploring alternate game designs that are based on community consensus rather than comparison to a gold-standard database.

The results from Dizeez provide evidence that online games can be used to help address the growing challenge of structured gene annotation. Through the game, we identified several novel gene-disease annotations that are well established in the literature, but not reflected in any public database. While the individual results presented here must be considered preliminary due to the small scale of this proof-of-concept experiment, they do hint at the tremendous potential of games for crowdsourcing annotation tasks in biology. Dizeez and other games for genetics and genomics can be played at http://genegames.org.

### Ethics Statement

Participants submitted answers through an online game interface. Informed consent was not required according to 45 CFR 46.101(b2) since no game players can be identified and disclosure of responses would result in no risk to participants.
