# Correction: Dizeez: An Online Game for Human Gene-Disease Annotation

**DOI:** 10.1371/annotation/efa93b55-c8f0-4943-b922-a585d68f0bd0

**Published:** 2013-08-26

**Authors:** Salvatore Loguercio, Benjamin M. Good, Andrew I. Su

There were errors in the Funding statement. The correct version of the Funding statement should read:

This research was supported by the National Institutes of Health (GM083924 and GM089820 to AIS), and benefitted from the NIH P50 Center of Excellence Grant to the San Diego Center for Systems Biology (SDCSB, GM085764). The funders had no role in study design, data collection and analysis, decision to publish, or preparation of the manuscript. 

